# Shared-Optics Dual-Port Interferometer for Simultaneous Displacement and Angular Motion Measurement

**DOI:** 10.3390/s26185920

**Published:** 2026-09-19

**Authors:** Gyoik Kim, June Gyu Park

**Affiliations:** Department of Astronomy, Yonsei University, 50 Yonsei-ro, Seodaemun-gu, Seoul 03722, Republic of Korea; gyoik_kim@yonsei.ac.kr

**Keywords:** dual-beam interferometer, cross-axis characterization, dual-channel measurement, multi degree of freedom measurement, IQ Interferometer, common path interferometer, balanced path interferometer

## Abstract

Conventional dual-beam interferometers typically provide a single interferometric phase observable, while simultaneous measurement of displacement and angular motion may require an additional optical path or auxiliary sensor. In this work, we develop a shared-optics dual-port interferometer for simultaneous displacement and angular-motion measurement without a substantial increase in optical complexity. The proposed configuration integrates two Michelson-like interferometers in a nearly common-path shared-optics architecture, using polarization-based routing to share key optical components while providing two independent phase readouts. Under a 120nm axial displacement at 70Hz, the two phase readouts exhibited closely matched responses and broadband phase levels at the $10^{-4}\;\mathrm{rad}/\sqrt{\mathrm{Hz}}$ level, while their difference reduced the shared displacement component to below 0.3% of the individual-channel response. In the simultaneous pitch and yaw excitation at 100 and 70Hz, the pitch- and yaw-driven responses were separated in the common and differential phase signals, while weak sum- and difference-frequency components associated with nonlinear and cross-axis actuator coupling were resolved at the $10^{-3}\;\mathrm{rad}/\sqrt{\mathrm{Hz}}$ level. These results demonstrate that the shared-optics architecture can extend a compact dual-beam interferometer from differential phase sensing to simultaneous displacement and angular-motion measurement, while retaining sufficient phase resolution to identify weak cross-axis actuator responses.

## 1. Introduction

Dual-beam interferometers use two spatially separated, typically parallel probe beams to obtain phase information from two locations or optical paths within the same measurement geometry. This parallel-beam geometry is useful for measuring differential optical path length changes, because perturbations that affect the two probe beams differently appear directly as a differential phase response [1,2,3]. As a result, dual-beam interferometers have been widely used for displacement, tilt, and vibration sensing [4,5,6]. However, the same differential geometry that improves robustness by canceling phase changes common to both probe beams does not directly provide separate access to the common phase response. This can limit the ability to distinguish common motion from differential motion in applications involving coupled mechanical dynamics or sensing under environmental disturbances.

Interferometric systems with multiple readout channels can provide complementary phase information through different sensitivities, reference conditions, or measurement geometries. Examples include dual-channel sensing and stabilization schemes using separate measurement and reference channels [7,8,9,10] and multi-axis interferometric arrangements for measuring multiple displacement or angular components in applications ranging from precision metrology [11,12,13,14,15,16,17] to test-mass sensing for drag-free control [18,19]. However, these approaches typically require additional sensing elements, specialized target optics, or independently aligned channels [20,21,22,23,24]. These additions can increase optical and mechanical complexity and may require additional attention to calibration, alignment, and stability.

In this work, we introduce a shared-optics dual-port interferometer (SDPI) that adds a second interferometric readout without duplicating the interferometer optics. The two interferometric channels are separated by polarization-based routing while sharing the principal optical components and nearly common optical paths. This shared configuration preserves the differential-sensing characteristics of a conventional dual-beam interferometer while providing an additional phase observable in a compact arrangement. The two phase readouts therefore provide simultaneous access to common and differential motion, which we use to demonstrate simultaneous displacement and angular-motion measurements and to resolve weak cross-axis actuator responses under combined actuation.

## 2. Design and Considerations

**Figure 1:**
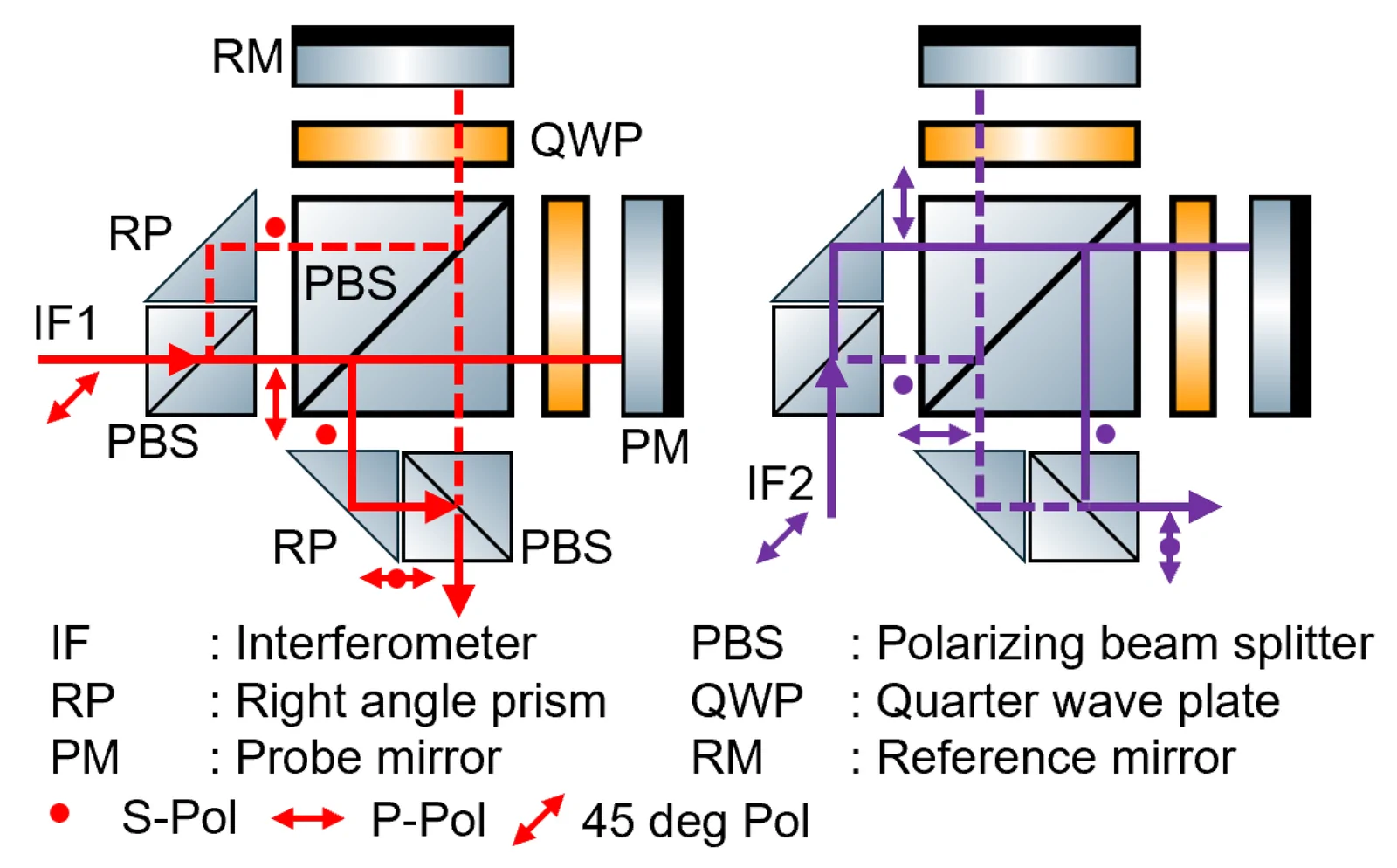
Schematic of the shared-optics dual-port interferometer (SDPI). IF1(red lines) and IF2(purple lines) denote the two input/output port paths, illustrated separately to clarify their beam routing while being implemented within a single shared-optics configuration. The symmetric and balanced layout allows IF1 and IF2 to share key optical components and follow nearly common optical routes while remaining optically isolated.

The shared-optics dual-port interferometer (SDPI) is based on a modified Mach–Zehnder interferometer configuration that realizes two phase readouts within a shared optical structure, as shown in Figure 1. Although the two port paths are illustrated separately to clarify their beam routing, they are implemented within a single shared-optics configuration. The two input/output port paths generate the interferometric readouts referred to as IF1 and IF2. The two paths originate from different input ports of the input polarizing beam splitter (PBS) in the modified Mach–Zehnder configuration. Each interferometric channel starts with a beam linearly polarized at $45^{\circ }$, which is separated into p- and s-polarized components by the input PBS. Because IF1 and IF2 have a symmetric optical configuration, the following description focuses on IF1, with the same principle applying to IF2.

The dashed line in Figure 1 denotes the beam reflected by the input PBS, which is initially the s-polarized component of the incident beam. This beam is directed to the right-angle prism (RP) and then propagates toward the mirror along the reflection path of the central PBS. After reflection at the mirror, the beam returns along the same path. During this round trip, it passes twice through the quarter-wave plate (QWP) oriented at $45^{\circ }$, converting its polarization from s to p. The converted p-polarized beam then follows the transmission paths of the central PBS and output PBS. The solid line in Figure 1 denotes the beam transmitted through the input PBS, which is initially the p-polarized component of the incident beam. This beam propagates toward the mirror along the transmission path of the central PBS. After reflection at the mirror, the beam returns along the same path. During this round trip, it passes twice through the QWP oriented at $45^{\circ }$, converting its polarization from p to s. The converted s-polarized beam is then reflected by the central PBS, redirected by the RP, and reflected by the output PBS, where it spatially overlaps with the converted p-polarized beam while remaining orthogonally polarized. This routing is chosen to maintain a balanced optical configuration between the two beams. The beams experience matched numbers of reflections and transmissions through the PBSs, as well as matched reflections at the RPs. One beam is reflected by the input PBS and input RP, reflected and transmitted at the central PBS, and transmitted through the output PBS. The other beam is transmitted through the input PBS, transmitted and reflected at the central PBS, reflected by the output RP, and reflected by the output PBS. Because the input side and output side PBS/RP components have the same size and optical characteristics, the two paths are well balanced except for the Michelson-like interferometric arms.

IF2 follows the symmetric counterpart of the IF1 path. In the shared input and output optics and at the central PBS, the beams from IF1 and IF2 spatially overlap only with orthogonal polarization states. This polarization orthogonality maintains optical isolation between the two interferometric channels while allowing them to share the same optical components. Although the interferometric arms in each channel have a Michelson-like configuration and are not fully balanced by themselves, the corresponding arms of IF1 and IF2 share the same mirror paths with equal optical lengths. This symmetry allows the two channels to serve as mutual references, so that imbalance from the Michelson-like arms can be reduced at the system level. Therefore, the SDPI combines nearly common optical paths in the shared-optics region with balanced channel routing, while the remaining arm-related imbalance can be further mitigated through mutual referencing between IF1 and IF2.

Polarization-based isolation also provides flexibility in the detection stage. The output beams of each interferometric channel remain in orthogonal polarization states, allowing different readout schemes to be implemented according to the target application without modifying the shared-optics interferometer itself. For example, the orthogonally polarized beams can be projected onto a common polarization axis for intensity-based interference readout. They can also be directed to a polarization-based quadrature detection module to obtain phase-shifted interference signals without altering the interferometer layout.

Careful implementation is still required in this configuration. Misalignment can disturb the optical-path and response balance between the two channels, reducing the correlation between their phase responses and increasing the residual mismatch between the two readouts. However, the shared-optics geometry helps maintain this mismatch at a manageable level through common optical components and symmetric channel routing. Finite polarization extinction can additionally introduce cross-talk between the two interferometric channels, modifying the signal offsets, amplitudes, and fringe visibility. Within the normal operating range of the quadrature readout, these effects primarily appear as residual errors in the reconstructed phase rather than as a change in the intrinsic phase-to-displacement conversion. For commercial polarization optics with extinction ratios on the order of 1000:1 or better, the leakage contribution is expected to remain small under the present operating conditions. In some applications, polarization-sensitive targets or optical paths may alter the polarization state. However, as discussed above, the effects of small polarization variations are mitigated by the high-extinction polarization optics and consequently appear only as small variations in the residual error, becoming relevant primarily in high-precision applications.

For compact implementation, the separate RP and PBS elements shown in Figure 1 can be replaced by PBS-coated lateral beam displacers (LBDs). In this form, the SDPI optical core requires only seven optical components: two LBDs, one central PBS, two QWPs, one probe mirror, and one reference mirror, and can be implemented within an approximately $100\times 100\;{\mathrm{mm}}^{2}$ footprint using standard low-profile optomechanics. All components are readily available as standard commercial elements, eliminating the need for custom-fabricated optics. The shared-optics components, including the LBDs, central PBS, and QWPs, can be integrated on a single mechanical platform, while the parallel-prism geometry of the LBDs provides passive alignment of the beam paths.

## 3. Experimental Setup

**Figure 2:**
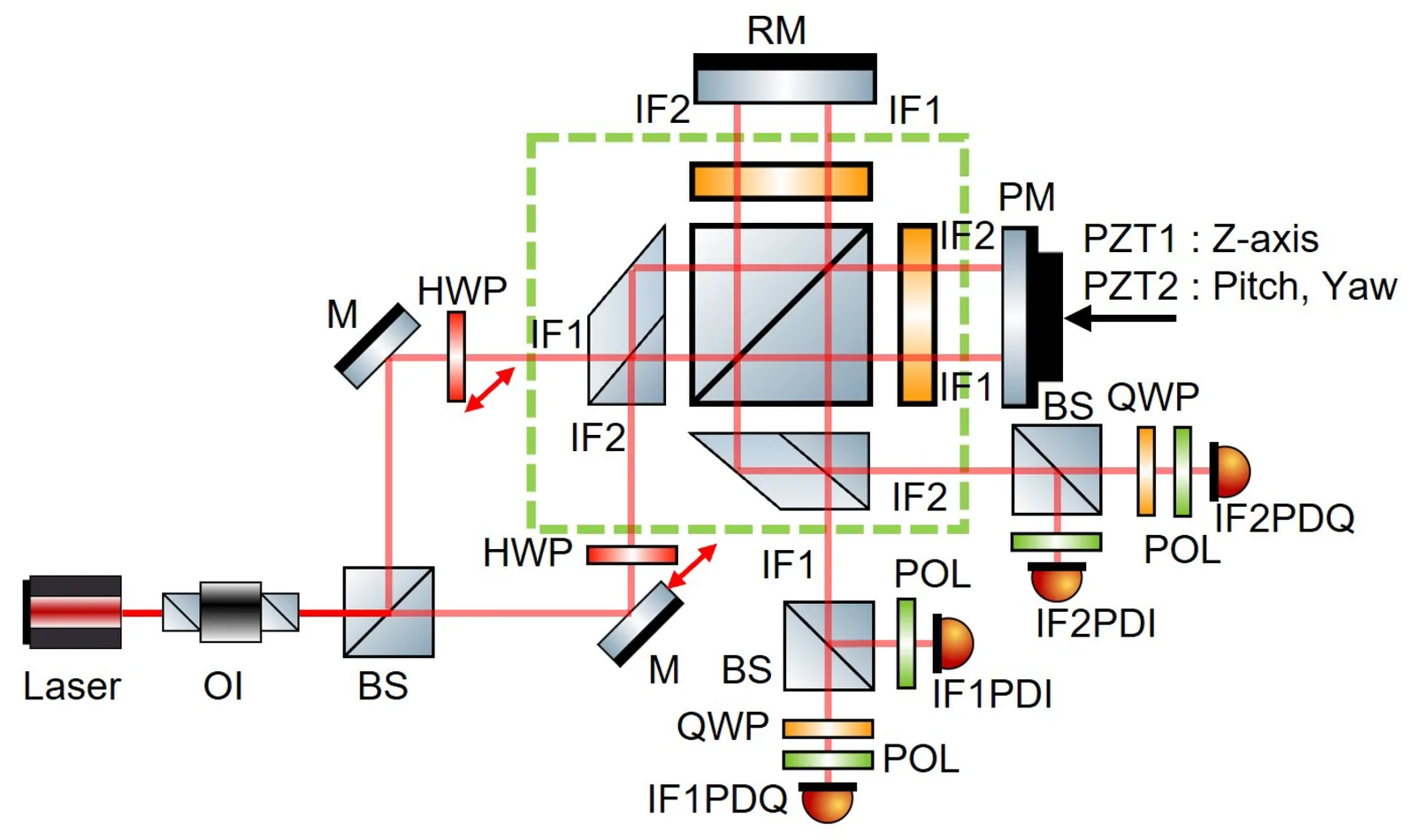
Experimental setup of the shared-optics dual-port interferometer (SDPI) for simultaneous displacement and angular-motion measurement. The SDPI module, indicated by the dashed green box, provides two correlated phase readouts for common and differential motion sensing. The output beams are measured using polarization-based quadrature detection modules, where PDI and PDQ denote the in-phase and quadrature photodetectors, respectively. Piezoelectric actuator (PZT1) actuates the target mirror along the axial direction, while PZT2 provides angular actuation. BS, beam splitter; OI, optical isolator; LBD, lateral beam displacer; RM, reference mirror; PM, probe mirror.

The experimental setup of the SDPI is shown in Figure 2. A stabilized 633 nm He-Ne laser (HRS015B, Thorlabs, Newton, NJ, USA) beam was divided into two beams. Each beam was linearly polarized at $45^{\circ }$ with respect to the PBS axes and injected into one of the two input ports of the SDPI, corresponding to IF1 and IF2. Although the present experiment uses two beams derived from a single laser source, the SDPI does not require a common optical source, and the two input ports can also be supplied independently according to the target application. At the output, each interferometric channel was measured using polarization-based quadrature detection with two photodiodes, denoted as PDI and PDQ, providing the in-phase and quadrature signals for phase extraction. The corresponding photodiode voltages, $V_{PDI}$ and $V_{PDQ}$, can be expressed using the Heydemann model [25], which includes DC offset, gain imbalance, and quadrature error.

(1) $$\begin{array}{cc} V_{PDI} & =E_{P}^{2}+E_{R}^{2}+2E_{P}E_{R}\cos\left(\phi_{P}-\phi_{R}\right)+p,\\ V_{PDQ} & =g\left[E_{P}^{2}+E_{R}^{2}+2E_{P}E_{R}\sin\left(\phi_{P}-\phi_{R}-\alpha \right)\right]+q \end{array}$$

where $E_{p}$ and $E_{R}$ are the magnitudes of the optical field amplitudes of the probe and reference beams, *p* and *q* denote the DC offsets, *g* is the channel gain imbalance, and α is the quadrature error. Because the experiment used a stabilized He-Ne laser and a mirror target with no intentional reflectivity change, the optical amplitudes $E_{P}$ and $E_{R}$ were assumed to remain nearly constant over each measurement. Thus, the offset terms in Equation (1) were treated as fixed parameters and removed numerically before phase conversion. Both the gain imbalance and quadrature error included in the Heydemann model were minimized at the hardware level, and no additional numerical correction for residual imbalances was applied in the present demonstration. The phase was then obtained from the offset-corrected PDI and PDQ signals using a direct arctangent operation.

**Figure 3:**
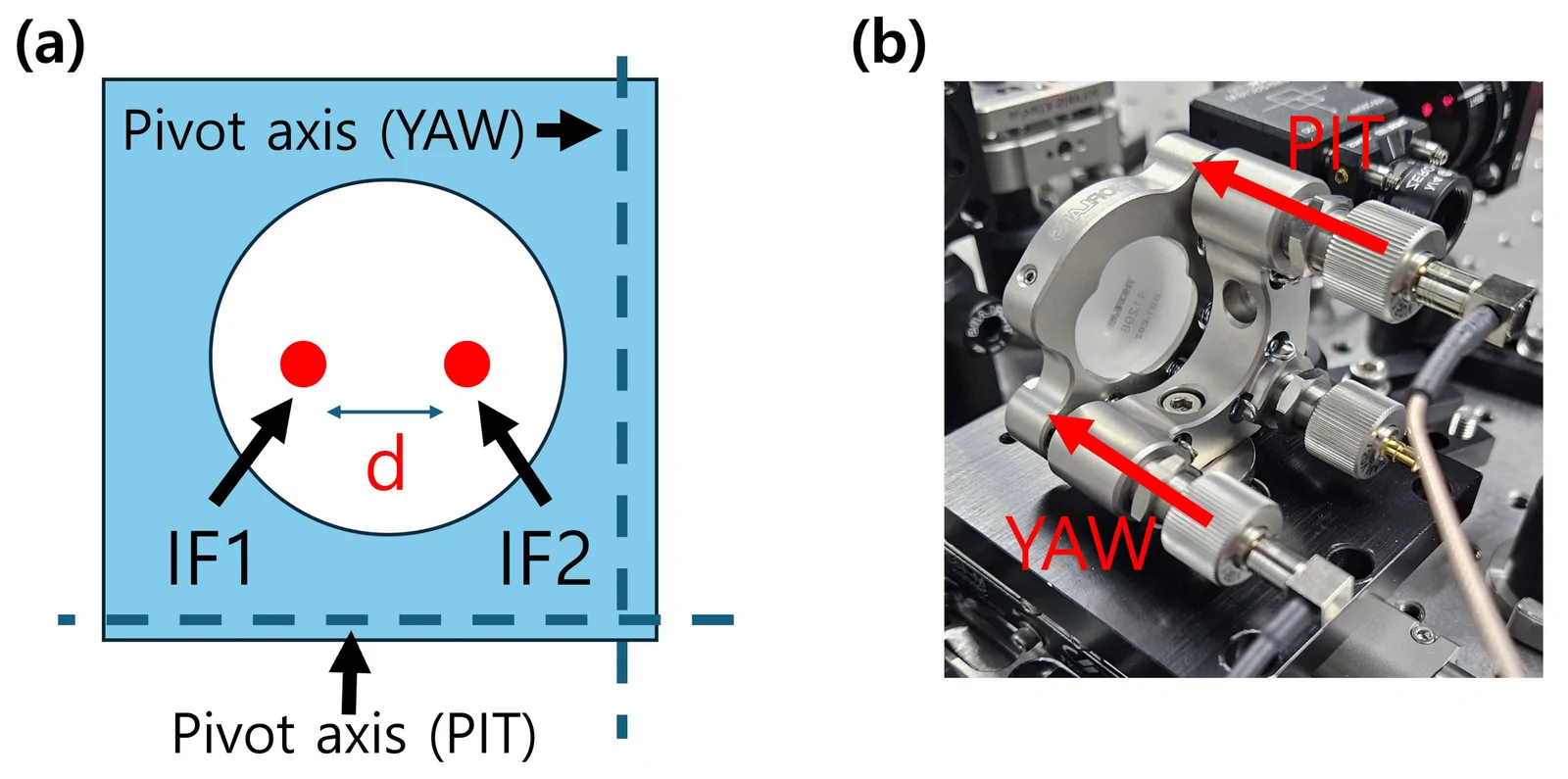
Actuator configuration for simultaneous displacement and angular-motion measurements. (**a**) Probing geometry in which the IF1 and IF2 probe beams are separated by a distance d on the target mirror. The target mirror is actuated by a piezo-driven *z*-axis translation stage for axial displacement and a piezo-actuated mirror mount for angular motion. (**b**) POLARIS-K1S3P piezo-actuated mirror mount showing the pitch and yaw actuator positions and corresponding pivot axes.

For simultaneous displacement and angular-motion measurements, an actuation assembly was installed in one arm of the SDPI, as shown in Figure 3. A piezo-actuated mirror mount (POLARIS-K1S3P, Thorlabs, Newton, NJ, USA) was placed on a piezo-driven *z*-axis translation stage to actuate the target mirror in axial displacement, pitch, and yaw. The IF1 and IF2 readout beams probed laterally separated positions on the mirror, with a separation distance d of 10mm set by the LBD geometry. Because of the displaced pivot axes of the mirror mount, pitch actuation produced nearly equal optical-path changes at the two sensing positions, whereas yaw actuation produced a relative optical-path difference. This geometry allowed axial and angular responses to be examined under combined actuation.

## 4. Experimental Results

**Figure 4:**
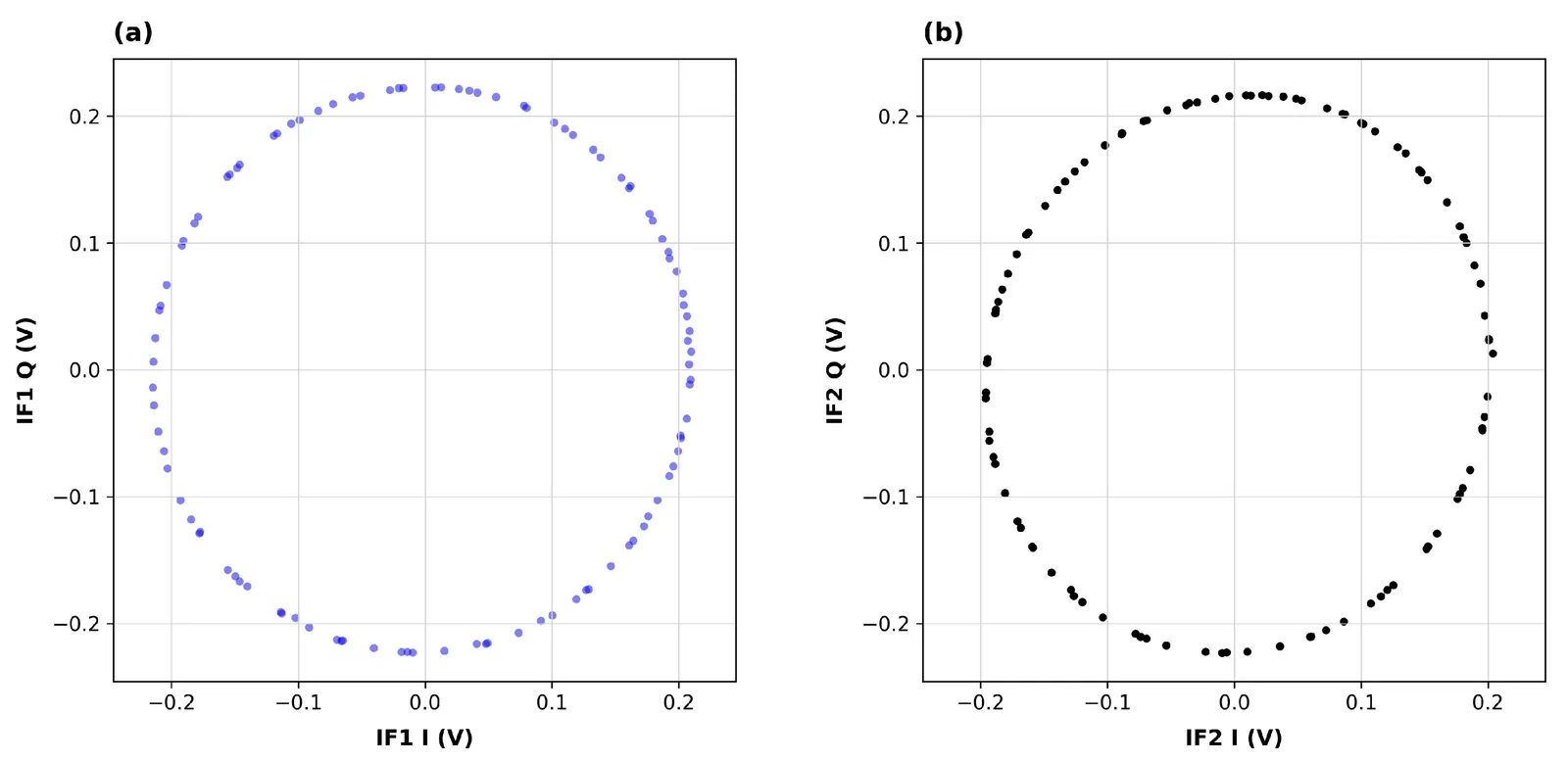
Representative Lissajous plots of the offset-corrected in-phase and quadrature signals used for phase extraction. (**a**) IF1 readout. (**b**) IF2 readout. The measured trajectories show the quadrature conditions used for direct arctangent phase extraction after offset correction. The residual cyclic phase errors had RMS values of 0.027rad for IF1 and 0.049rad for IF2, corresponding to 0.43% and 0.78% of a 2π phase cycle, respectively.

We first examined the quadrature signal condition used for phase extraction in the two interferometric readouts. Although a four-channel balanced quadrature scheme could be used [26], the present experiment used two-channel in-phase and quadrature detection because the optical amplitudes were expected to remain nearly constant over each measurement. Only the DC offsets were numerically corrected before direct arctangent phase conversion, while residual gain and quadrature imbalance were minimized through optical alignment and adjustment of the I/Q signal balance. Figure 4 shows the resulting Lissajous trajectories for IF1 and IF2. The trajectories are close to circular, with only slight residual ellipticity and quadrature imbalance. The corresponding cyclic phase errors were 0.027rad RMS for IF1 and 0.049rad RMS for IF2, corresponding to 0.43% and 0.78% of a 2π phase cycle, respectively. These values indicate that the remaining I/Q nonidealities were sufficiently small for direct arctangent phase extraction in the present measurements. The trajectories were obtained using a 100mHz slow ramp applied to PZT1 for *z*-axis displacement. The nonuniform point spacing results from the nonlinear piezo response, which produces a nonuniform phase sweep during the ramp.

Based on the quadrature phase readout described above, we measured the response of the SDPI to common-mode displacement (CMOD). Referring to the experimental setup in Figure 3, CMOD was applied using PZT1 (piezoelectric actuator), which was mounted on the translational stage and actuated the target mirror along the *z*-axis. Under CMOD, both interferometric readouts should follow the same displacement-dependent phase variation, while each readout retains its own interferometric response. This measurement provides a direct test of whether the shared-optics dual-port arrangement provides correlated common phase readout for the same axial motion [9,27].

**Figure 5:**
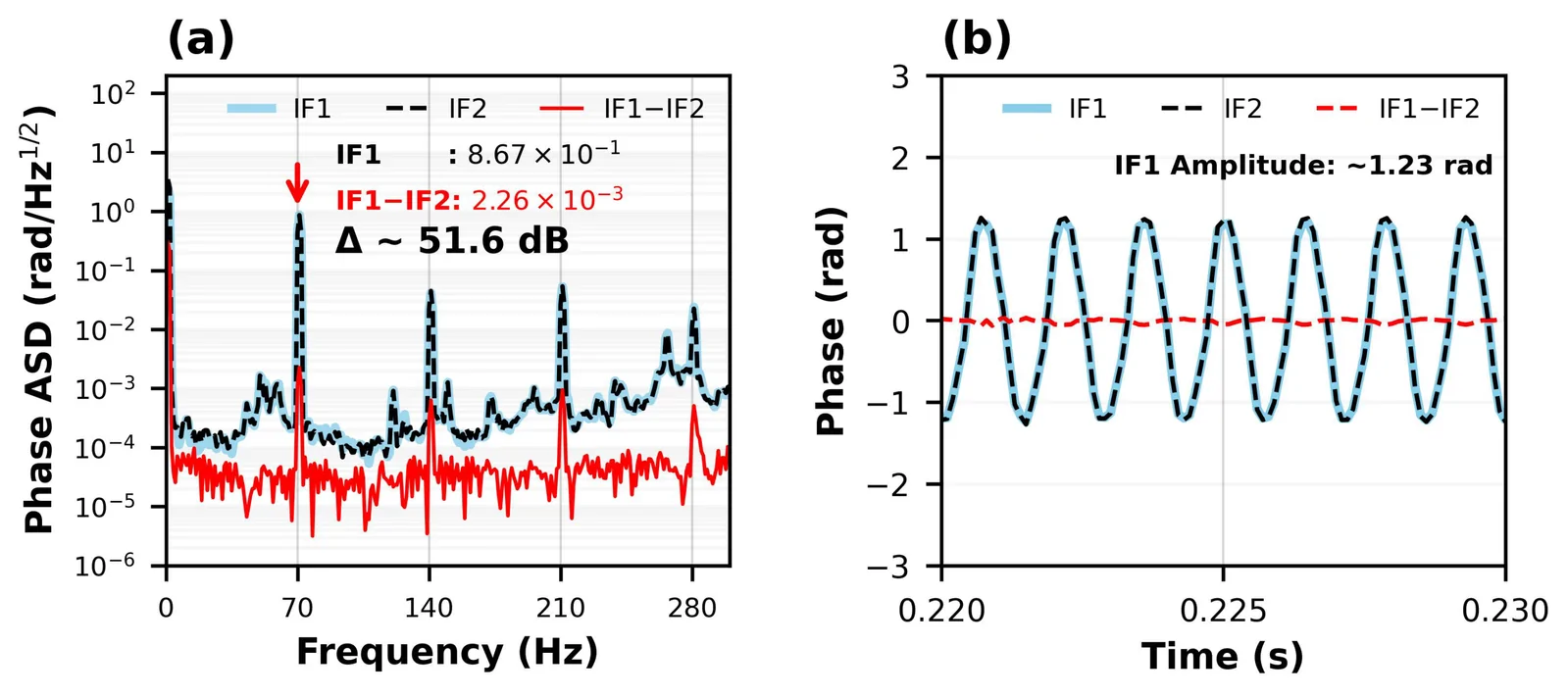
Common-mode displacement (CMOD) response under 70Hz axial mirror actuation. (**a**) Phase amplitude spectral density (ASD) of IF1 (sky-blue), IF2 (black dashed), and the differential phase IF1–IF2 (red). The 70Hz CMOD component is measured by both interferometric readouts and is suppressed by 51.6dB in the differential phase. (**b**) Representative time-domain phase responses measured under the same actuation condition, showing nearly identical phase variations in IF1 and IF2 and a small residual differential phase, with the measured phase amplitude consistent with the applied axial displacement.

For the CMOD measurement, a sinusoidal displacement of approximately 120 nm was applied at a driving frequency of 70Hz. The IF1 and IF2 quadrature signals were simultaneously digitized using four input channels of a 20-bit DAQ module (USB-6451, National Instruments, Austin, TX, USA). Figure 5a presents the amplitude spectral density (ASD) of the measured phase responses, while Figure 5b shows representative time-series data. The time-series data provide a direct comparison of the two readouts, showing that IF1 and IF2 follow nearly identical phase variations under the applied CMOD while only a small residual remains in their differential response. Since each interferometric channel operates in a reflection geometry, the displacement, Δx, is related to the measured phase by Δx=(λ/4π)Δϕ. The measured phase amplitude of approximately 1.23rad therefore corresponds to a peak-to-peak mirror displacement of 124nm, which is consistent with the nominal applied displacement of approximately 120nm within the expected variation in the open-loop piezo actuation. The frequency- and time-domain data were acquired in separate runs and are presented to show the common-mode response in the frequency and time domains, respectively. The ASD spectra in Figure 5a were obtained by averaging ten consecutive 1s power spectral density (PSD) measurements. The averaged PSD was then converted to ASD, yielding phase spectra in units of $\mathrm{rad}/\sqrt{\mathrm{Hz}}$. This averaging reduces the statistical variance of the PSD estimate, but should not be interpreted as an actual reduction in the underlying measurement noise floor [28].

Figure 5a shows a clear peak at the 70Hz CMOD driving frequency, with weaker harmonic components also visible at higher frequencies (140Hz, 210Hz and 280Hz). The IF1 and IF2 spectra exhibit closely matched responses under the common-mode actuation. To distinguish the nearly overlapping traces, IF1 is plotted as a thick translucent sky-blue solid line, while IF2 is plotted as a black dashed line. At the 70Hz driving frequency, the residual differential phase IF1–IF2 is strongly reduced relative to the IF1 response, corresponding to 51.6dB suppression, with the residual differential amplitude reduced to below approximately 0.3% of the IF1 response. The suppression was calculated from the ASD amplitude ratio as $20\log_{10}\left(A_{\text{IF}1}/A_{\text{IF}1-\text{IF}2}\right)$, where $A_{\text{IF}1}$ and $A_{\text{IF}1-\text{IF}2}$ denote the ASD amplitudes of IF1 and IF1–IF2 at the 70Hz driving peak, respectively. The suppression was calculated using the conventional amplitude-ratio definition in decibels. Since the achievable suppression is strongly influenced by alignment-induced imbalance between the two interferometric readouts, the channels were carefully adjusted to balance their responses. This adjustment primarily affects the path and response matching between the two channels, whereas the basic displacement and angular conversions remain primarily determined by the interferometer geometry and optical wavelength. Under these conditions, suppression above 40dB was consistently observed in the tested CMOD measurements.

The harmonic components were also reduced in the differential phase, although with different suppression levels from the fundamental component. This behavior indicates that a substantial part of the harmonic response is shared between IF1 and IF2. The remaining differential component is attributed to small residual mismatches between the two readouts. Despite these residual differences, the IF1–IF2 spectrum remains substantially below the individual IF1 and IF2 spectra. At the 70 Hz driving peak, the residual differential phase is at the $10^{-3}\;\mathrm{rad}/\sqrt{\mathrm{Hz}}$ level, corresponding to a sub-nanometer optical path equivalent at 633nm. Considering that the present setup uses standard commercial optical components, including LBDs with dimensional tolerances on the order of 0.1mm, further improvement in suppression may be possible through tighter component tolerances and integration of the shared optics on a single mechanical platform.

The broadband phase spectra in Figure 5a show a similar behavior. The individual IF1 and IF2 readouts exhibit nearly identical broadband noise spectra at the $10^{-4}\;\mathrm{rad}/\sqrt{\mathrm{Hz}}$ level over much of the measured bandwidth, corresponding to a mirror-displacement equivalent of approximately $5\;\mathrm{pm}/\sqrt{\mathrm{Hz}}$ at 633nm. Their differential phase is reduced to an approximately flat residual level, reaching the $10^{-5}\;\mathrm{rad}/\sqrt{\mathrm{Hz}}$ range over part of the bandwidth. For the 10mm beam separation used in the present geometry, this residual corresponds to an angular-motion equivalent of approximately $0.05\;\mathrm{nrad}/\sqrt{\mathrm{Hz}}$. The geometric relation used for this angular-motion conversion is discussed later. The close correspondence between the two individual spectra and their reduction after differential subtraction indicate that a substantial fraction of the measured broadband fluctuations originates from perturbations common to both readouts, such as environmental or system-level disturbances, rather than from independent noise intrinsic to each interferometric channel. This common-response behavior may also be useful in dual-channel schemes for environmental disturbance compensation or correlated reference sensing. Minor spectral components, including those near the power-line frequency and other laboratory perturbation frequencies, are also reduced in the differential phase, further supporting this interpretation. The remaining broadband differential floor is consistent with the effective readout floor of the present measurement system, including photodetection and electronic readout contributions as well as the finite resolution of the 20-bit DAQ.

The IF1 and IF2 readouts provide displacement measurements at two spatially separated sensing positions, while the relative displacement between the two positions can be converted to mirror tilt through the measurement geometry. This relation enables displacement and angular motion to be obtained simultaneously from the two phase readouts. Figure 3b shows the yaw-actuation configuration used to evaluate this relation experimentally. The two probe beams are incident on spatially separated positions of the same probe mirror. From the optical layout in Figure 1, angular motion of the probe mirror affects only the optical paths associated with reflection from the PM, while the rest of the interferometer remains unchanged. Because the geometrical path changes are nearly symmetric between the two channels, the effect of higher-order geometrical terms on the phase-to-displacement conversion is expected to be reduced in the differential response. Under the small-angle approximation, the longitudinal displacement at each sensing position can be expressed as an arbitrary common longitudinal displacement and a tilt-induced displacement, with higher-order terms in the angular motion retained separately. Let $\Delta x_{1}$ and $\Delta x_{2}$ denote the longitudinal displacements measured at the IF1 and IF2 sensing positions, respectively, and let $r_{1}$ and $r_{2}$ denote their distances from the kinematic pivot. The two displacement responses can then be written as

(2) $$\Delta x_{1}=\Delta x_{0}+r_{1}\theta +O_{1}\left(\theta^{2}\right),\;\Delta x_{2}=\Delta x_{0}+r_{2}\theta +O_{2}\left(\theta^{2}\right),$$

where $\Delta x_{0}$ represents an arbitrary common longitudinal displacement of the probe mirror, θ is the angular displacement, and $O_{1}\left(\theta^{2}\right)$ and $O_{2}\left(\theta^{2}\right)$ denote higher-order terms associated with the angular motion in the two channels. Taking the difference between the two displacement responses gives

(3) $$\Delta x_{1}-\Delta x_{2}=\left(r_{1}-r_{2}\right)\theta +\left[O_{1}\left(\theta^{2}\right)-O_{2}\left(\theta^{2}\right)\right].$$

As discussed above, the angular-motion-induced higher-order path changes associated with the probe mirror are nearly symmetric in the two channels, with similar magnitude and direction. Their differential contribution is therefore strongly reduced, and the remaining correction can be neglected under the small-angle condition. The relation then reduces to

(4) $$\Delta x_{1}-\Delta x_{2}≃\left(r_{1}-r_{2}\right)\theta .$$

With the separation between the two sensing positions defined as $d=r_{1}-r_{2}$, the angular displacement is given by

(5) $$\theta =\frac{\Delta x_{1}-\Delta x_{2}}{d}.$$

Since each interferometric channel operates in a reflection geometry, $\Delta x_{i}=\left(\lambda /4\pi \right)\Delta \phi_{i}$. The angular displacement can therefore be expressed directly in terms of the two measured phases as

(6) $$\theta =\frac{\lambda }{4\pi d}\left(\Delta \phi_{1}-\Delta \phi_{2}\right).$$

**Figure 6:**
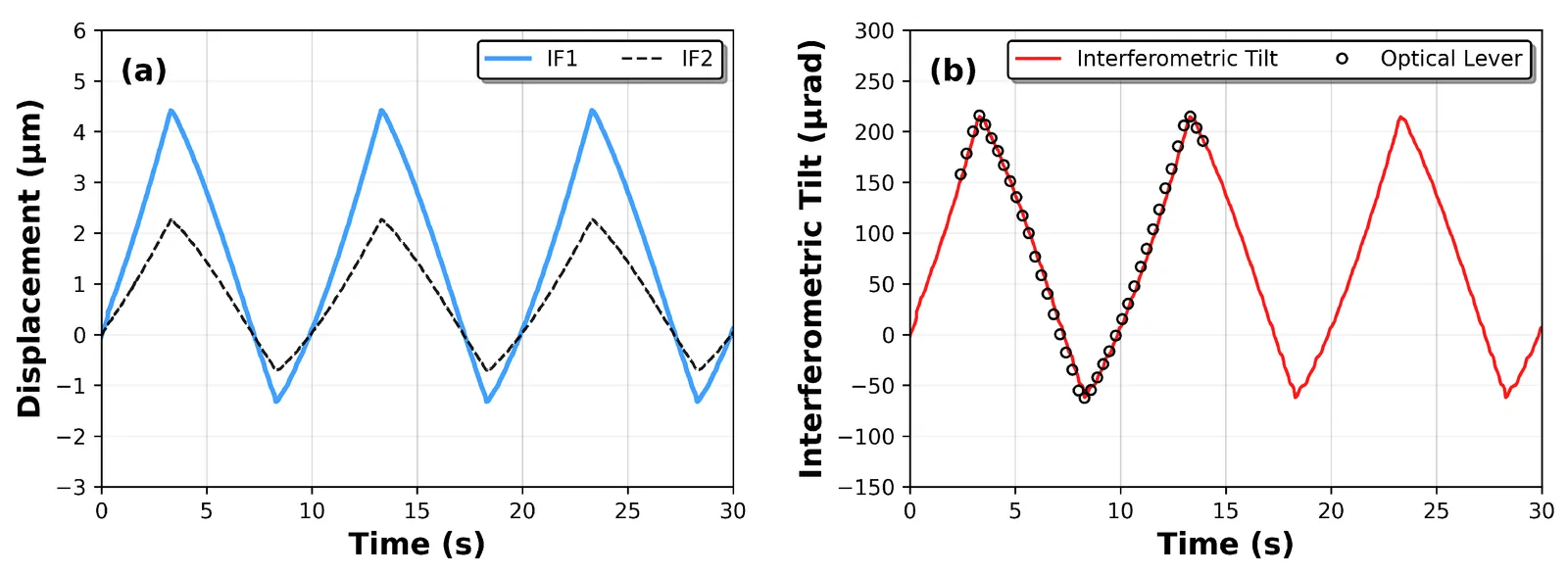
Simultaneous displacement and angular-motion measurement under yaw actuation. (**a**) Time-domain displacement responses at the two sensing positions obtained by converting the IF1 and IF2 phase changes using a wavelength of 633nm. (**b**) Mirror tilt estimated from the relative displacement between IF1 and IF2 using the geometric relation for the 10mm beam separation, together with an independent optical-lever measurement obtained under the same actuation conditions and shown by the open-circle markers.

Equation (6) is equivalent to the conventional angular relation used in dual-beam interferometry [29], in which the differential phase is converted to angular motion through the separation between the two sensing positions. This relation shows that the angular measurement is determined primarily by the optical wavelength (λ), the separation between the two sensing positions (*d*), and the differential phase between the two channels. Accordingly, the primary uncertainty contributions to the angular measurement arise from the differential phase determination and the geometrical scale factor defined by the beam separation, with a frequency-stabilized laser used as the optical source. Figure 6 shows the experimental results obtained using this tilt measurement relation.

Yaw motion was applied to the probe mirror, and the displacement responses at the two sensing positions were measured by IF1 and IF2. Because the PZT controller provided only a positive drive voltage in this measurement, the initial mirror orientation was intentionally offset from the nominal alignment condition, and the yaw scan was performed about a tilted operating point. A relatively large yaw actuation was used both to avoid a very small-signal condition, in which the open-loop piezo response can be more affected by hysteresis and other nonlinearities, and to provide a sufficiently large angular excursion for independent optical-lever measurement. A 0–10V sawtooth signal at 100mHz was applied to the PZT controller (MDT 693A, Thorlabs, Newton, NJ, USA) with a gain of 7.5, resulting in a 0–75V drive range at the piezo actuator.

As shown in Figure 6a, the phase responses measured by IF1 and IF2 were converted to the corresponding mirror displacements using Δx=(λ/4π)Δϕ, with λ=633nm. The measured peak-to-valley displacements were 5.74 µm for IF1 and 2.97 µm for IF2, giving a displacement ratio of 1.93. From Equation (4), this ratio corresponds to $r_{1}/r_{2}$, while the known separation between the two sensing positions gives $r_{1}-r_{2}=d=10\;\mathrm{mm}$. These relations yield effective pivot distances of approximately $r_{1}=20.7\;\mathrm{mm}$ and $r_{2}=10.7\;\mathrm{mm}$. These inferred pivot distances are consistent with the pivot location estimated from the mechanical drawing of the POLARIS-K1S3P piezo mirror mount and the current beam positions on the probe mirror.

Figure 6b shows the mirror tilt obtained by converting the displacement responses in Figure 6a using Equation (6). The reconstructed interferometric tilt exhibits a peak-to-valley angular excursion of 277μrad and clearly shows the hysteretic tendency associated with the open-loop piezo actuation. This value is broadly consistent with the specified angular range of the Thorlabs POLARIS-K1S3P mirror mount under the applied drive condition. However, because the open-loop piezo response can vary between individual devices, an optical lever was used for an independent quantitative comparison. The optical-lever measurements are shown as open-circle markers in Figure 6b. The optical lever was configured in reflection geometry with a charged-coupled device (CCD) camera positioned 2.5m from the mirror. A relatively long baseline was used to improve the angular resolution of the optical lever by increasing the beam displacement on the CCD for a given mirror tilt. Since the optical lever path could not be integrated into the present interferometer setup because of spatial constraints, the mirror mount was tested separately under the same actuation conditions, and the corresponding beam displacement was measured using the CCD. The optical-lever measurement yielded a peak-to-valley angular excursion of 279 ± 3 µrad, where the estimated uncertainty includes contributions from the CCD beam-center determination, the mirror-to-camera distance, and beam-position variation on the mirror. The optical-lever measurement agrees with the interferometrically reconstructed tilt within the estimated uncertainty.

For the interferometric tilt reconstruction, uncertainty in the beam separation *d* directly affects the angular scale factor in Equation (6). In the present setup, the angular conversion was initially evaluated using a nominal beam separation of d=10mm. The commercial LBD has a dimensional tolerance of approximately ± 100 µm, corresponding to an angular scale uncertainty of about 1%. A direct measurement of the beam separation gave d=10.0±0.05mm, reducing the corresponding scale uncertainty to approximately 0.5%. Based on the differential phase noise level shown in Figure 5, a phase sensitivity on the order of $10^{-4}\;\mathrm{rad}$ is achievable under the present operating conditions, corresponding to 0.0016% of a 2π phase cycle. Therefore, if angular measurements are to fully exploit the available phase sensitivity, the beam separation *d* would need to be calibrated to a comparable relative level. For d=10mm, this corresponds to approximately 0.16 µm, which is experimentally feasible with appropriate calibration. At larger angular excursions, the reflected-beam geometry departs further from the nominal alignment condition, which can increase the imbalance between the quadrature signals and thereby enhance cyclic errors in the reconstructed phase. Under the nominal alignment condition, the residual cyclic errors shown in Figure 4 are 0.43% and 0.78% of a 2π phase cycle for IF1 and IF2, respectively. As the excursion increases, this imbalance can become more pronounced, causing the cyclic phase error to grow to the percent level, which accounts for the small residual distortions near the peak and valley of the reconstructed tilt in Figure 6b. Therefore, precision angular measurement requires accurate calibration of geometrical parameters, including the beam separation *d* and sensing-beam parallelism, and operation within a dynamic range that avoids significant growth of alignment-dependent cyclic errors.

**Figure 7:**
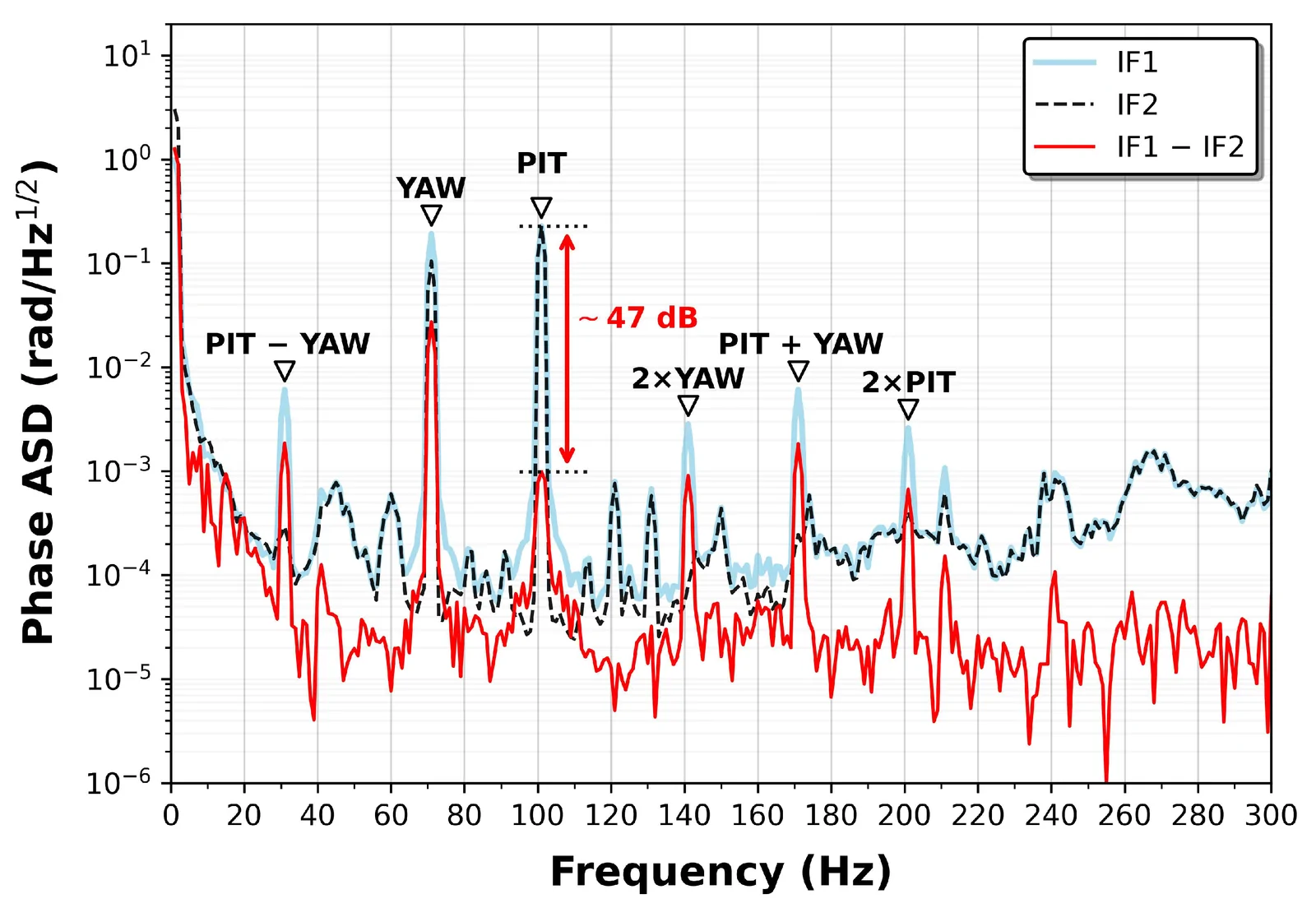
Frequency-domain phase responses of IF1, IF2, and IF1–IF2 under simultaneous pitch and yaw actuation. The pitch actuation at 100Hz produces a mainly common-mode phase response in IF1 and IF2 and is therefore suppressed in the differential phase. In contrast, the yaw actuation at 70 Hz produces a differential phase response associated with target tilt and remains visible in IF1–IF2. In the figure annotations, PIT and YAW denote the pitch- and yaw-drive frequencies, respectively; PIT − YAW and PIT + YAW indicate the difference- and sum-frequency components, while 2×YAW and 2×PIT denote the corresponding second harmonics.

Having established the displacement-to-tilt conversion and its practical limitations under yaw actuation, we next examined the response of the SDPI under simultaneous pitch and yaw excitation. This simultaneous excitation also provides a practical means of observing cross-axis motion of the piezo-actuated mirror. The actuator configuration introduced in Figure 3 was used for this measurement. Because the two sensing beams were laterally separated on the mirror, pitch actuation produced mainly a common-mode phase response, whereas yaw actuation produced a differential phase response associated with target tilt. To distinguish the two motion components in the measured signals, the pitch and yaw actuators were driven at different frequencies and analyzed in the frequency domain. The pitch and yaw actuators were driven at 100Hz and 70Hz, respectively, using sinusoidal signals generated by a function generator. Each signal had a peak-to-peak voltage of 200mV and was amplified by a factor of 7.5 using a piezo controller (MDT 693A, Thorlabs, Newton, NJ, USA) before being applied to the corresponding actuator. Figure 7 shows the ASD spectra of the measured phase responses under simultaneous pitch and yaw actuation. IF1, IF2, and the differential phase IF1–IF2 are plotted as a sky-blue solid line, a black dashed line, and a red solid line, respectively.

Although the 100Hz and 70Hz components appear with similar amplitudes in the individual IF1 and IF2 spectra, their behavior in the differential readout is clearly different. The 100Hz component originates from the pitch actuation and appears nearly equally in IF1 and IF2, consistent with a predominantly common displacement response. Consequently, this component is strongly reduced in IF1–IF2. In contrast, the 70Hz yaw component remains clearly visible in the differential phase because the angular motion produces different displacements at the two spatially separated sensing positions. The pitch and yaw responses can therefore be identified simultaneously in the measured spectra. Their distinct common-mode and differential responses allow the pitch- and yaw-induced motions to be distinguished in the frequency domain.

The spectra also reveal several weaker components associated with the coupled motion of the piezo-actuated mirror. In particular, peaks near 30Hz and 170Hz correspond to the difference and sum frequencies of the 100Hz pitch and 70Hz yaw excitations, respectively. These components were not observed under individual pitch or yaw excitation and shifted with the corresponding difference and sum frequencies when the driving frequencies were changed, indicating intermodulation during simultaneous actuation. Although similar spectral peaks may also appear in a conventional parallel-beam interferometer, a differential signal alone does not readily distinguish whether such weak components originate from actual relative motion or from other channel-dependent effects. In the shared-optics configuration, the closely matched individual readouts allow the common and differential contributions of these weak components to be directly compared, helping distinguish motion-related nonlinear and cross-axis responses from other channel-dependent effects. The component near 200Hz corresponds to the second harmonic of the 100Hz pitch excitation. In contrast to the CMOD case in Figure 5, where the harmonic components were largely common to IF1 and IF2 and were therefore reduced in the differential readout, the 200Hz component remains clearly visible in IF1–IF2. This indicates that the second-harmonic response includes a differential motion component. In the actuator geometry of Figure 3, this behavior is consistent with cross-axis motion associated with the kinematic coupling of the piezo-actuated mirror mount. These weaker spectral components demonstrate that the shared-optics differential readout can resolve nonlinear and cross-axis motions of the piezo-actuated mirror during simultaneous multi-axis excitation. In addition, no prominent spectral components are observed in the differential readout other than those that can be associated with the applied excitations, their harmonics, or intermodulation products. Polarization leakage can degrade fringe visibility or introduce channel-dependent phase errors, potentially producing complex spectral features through channel-dependent polarization mixing and phase reconstruction. Since these responses are not necessarily correlated between IF1 and IF2, they would not be expected to cancel efficiently in the differential readout. Such effects can contribute to the residual response and affect the differential rejection, but the spectra in Figure 7 show no clear evidence of a significant polarization-leakage influence, suggesting that it is not a major contributor under the present measurement conditions. Overall, the observed differential components are on the order of $10^{-3}\;\mathrm{rad}/\sqrt{\mathrm{Hz}}$, corresponding to a relative-displacement equivalent of approximately $50\;\mathrm{pm}/\sqrt{\mathrm{Hz}}$ or an angular-motion equivalent of approximately $5\;\mathrm{nrad}/\sqrt{\mathrm{Hz}}$ for the 10mm beam separation.

## 5. Conclusions

In this work, we demonstrated a shared-optics dual-port interferometer for simultaneous displacement and angular-motion measurement using two spatially separated phase readouts. Polarization-based routing in a symmetric layout generates two correlated interferometric signals that share the principal optical components. The compact interferometric core consists of seven standard optical elements—two LBDs, one PBS, two QWPs, one probe mirror, and one reference mirror—all of which are available as standard commercial optics. The source and detection units can be adapted to the target application, for example using a fiber-delivered source or a modular detector unit.

The common-mode displacement experiment confirmed that the shared-optics configuration provides closely matched responses to the same axial mirror motion. At the 70Hz driving frequency, the residual differential response was below approximately 0.3% of the IF1 response. The individual readouts exhibited broadband phase levels on the order of $10^{-4}\;\mathrm{rad}/\sqrt{\mathrm{Hz}}$, corresponding to a mirror-displacement equivalent of approximately $5\;\mathrm{pm}/\sqrt{\mathrm{Hz}}$. The differential residual reached the $10^{-5}\;\mathrm{rad}/\sqrt{\mathrm{Hz}}$ range over part of the measured bandwidth, corresponding to an angular-motion equivalent of approximately $0.05\;\mathrm{nrad}/\sqrt{\mathrm{Hz}}$.

Under simultaneous multi-axis actuation, the shared-optics readout distinguished pitch-induced common displacement from yaw-induced differential motion. The differential response also revealed weak nonlinear and cross-axis components associated with the kinematic structure of the piezo mirror mount, providing insight into its coupled mechanical behavior. These components were observed at differential phase levels on the order of $10^{-3}\;\mathrm{rad}/\sqrt{\mathrm{Hz}}$, corresponding to relative-displacement and angular-motion equivalents of approximately $50\;\mathrm{pm}/\sqrt{\mathrm{Hz}}$ and $5\;\mathrm{nrad}/\sqrt{\mathrm{Hz}}$, respectively.

The observed performance suggests potential applications in precision motion systems where a relatively large common displacement coexists with much smaller differential or angular responses. A representative example is precision stage characterization, where translational motion, weak tilt, and coupled or cross-axis dynamics may need to be resolved simultaneously. This capability may also be useful for characterizing coupled mechanical dynamics in actuators and mounts. In addition, although the present study focuses on displacement and angular-motion measurements, simultaneous access to common and differential phase responses may also be useful in sample–reference measurements, such as refractive-index or concentration monitoring, where common phase variations associated with environmental disturbances need to be distinguished from smaller target-induced phase responses.

## Data Availability

Data underlying the results presented in this paper are not publicly available at this time but may be obtained from the authors upon reasonable request.
